# Supplementary material for: Quantification of Airborne Particulate Matter and Trace Element Deposition on Hedera helix and Senecio cineraria Leaves
Source: Plants (Basel). 2024 Sep 7;13(17):2519. doi: 10.3390/plants13172519 (PMC11397657; doi:10.3390/plants13172519)
Supplement: Supplementary file 1 [file plants-13-02519-s001.zip › plants-3132764-supplementary.pdf]

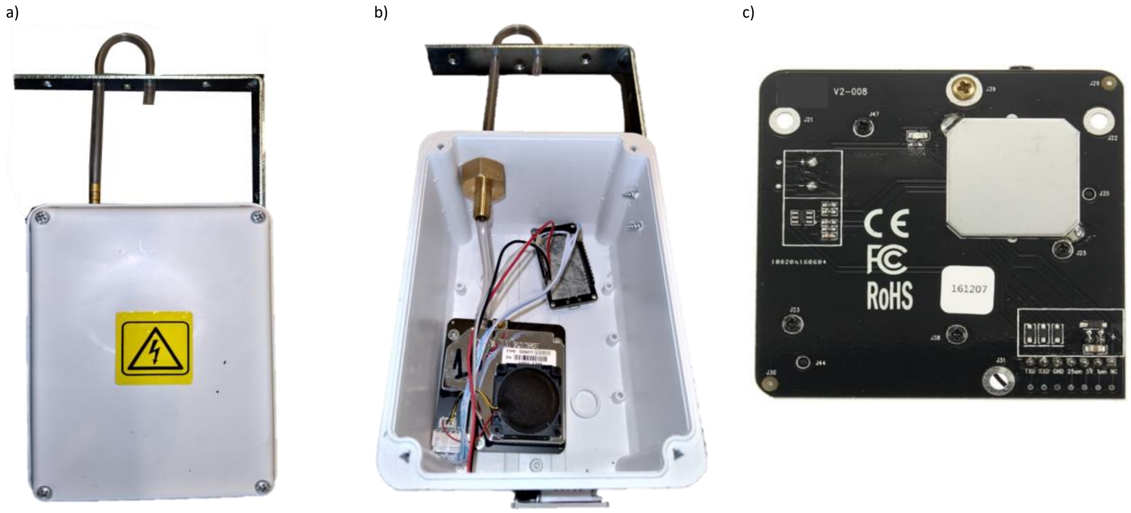

Figure S1. Particulate matter monitoring station. (a) Operative monitoring station; (b) Inside monitoring station; (c) Detail of matriculate matter sensor (Nova fitness SDS011).

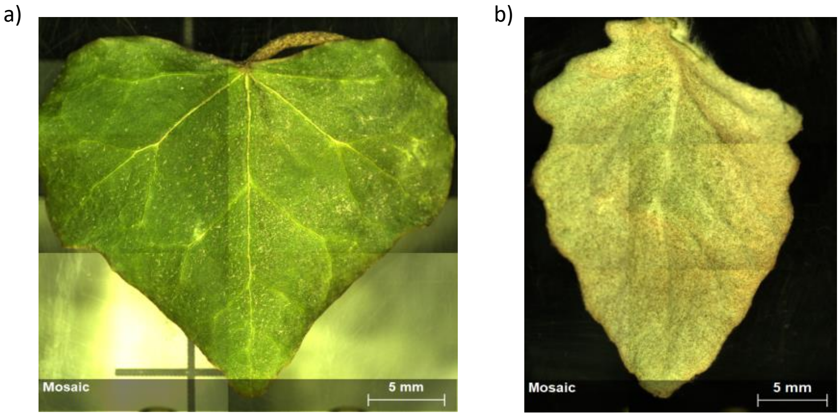

Figure S2. Image acquired by XRF camera applying background correction and reduced image range. (a) *Hedera helix*; (b) *Senecio cineraria*.

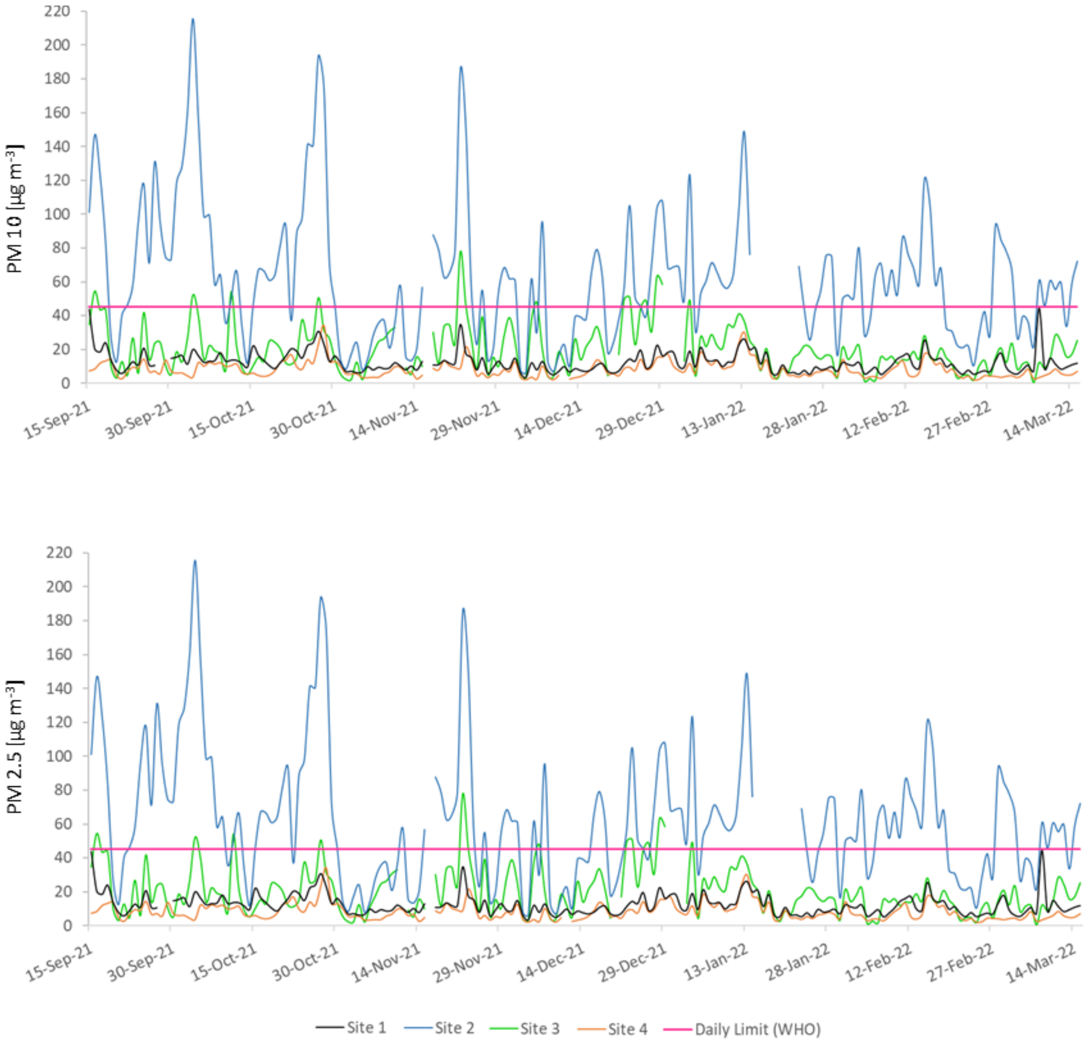

Figure S3. Daily average concentrations of PM 10 and PM 2.5 recorded at each site. Pink line represents the WHO annual threshold (PM 10= 15 µg m^{-3}; PM2.5= 5 µg m^{-3}).

Table S1. Semi quantitative element concentrations Normalized weight % in leaf tissue by XRF.

Plant	Site	Time	Na	Mg	Al	Si	P	S	k	Ca	Ti	Mn	Fe	Zn
Hedera helix	Site 1	0m	0.30±0.30	2.26±1.25	2.73±1.87	15.60±8.58	1.93±0.36	4.03±1.21	35.54±14.27	36.30±4.88	0.16±0.15	0.34±0.39	0.76±0.47	0.04±0.03
		3m	2.17±0.89	1.97±1.59	2.72±2.08	14.71±11.93	1.51±0.41	3.39±0.74	32.70±13.92	38.94±5.77	0.23±0.21	0.31±0.23	0.97±0.27	0.37±0.06
		6m	2.18±0.89	2.06±1.61	2.74±2.08	14.82±11.98	1.55±0.41	3.41±0.73	32.90±13.75	39.24±5.77	0.23±0.22	0.32±0.24	1.00±0.29	0.38±0.06
	Site 2	0m	0.52±0.64	2.27±0.94	2.81±1.37	16.47±7.44	2.70±1.18	4.66±1.42	25.77±9.69	43.23±13.26	0.16±0.09	0.46±0.25	0.88±0.55	0.05±0.02
		3m	0.92±0.94	1.73±0.98	3.61±1.31	12.29±5.23	1.44±0.53	2.48±1.98	39.82±10.10	35.72±8.83	0.15±0.09	0.11±0.09	1.35±0.42	0.34±0.55
		6m	0.20±0.23	0.70±0.54	10.69±1.98	34.74±7.83	0.42±0.17	1.27±0.30	18.65±5.63	29.61±4.69	0.37±0.10	0.26±0.21	3.07±0.34	0.02±0.02
	Site 3	0m	0.29±0.37	2.53±1.06	2.24±0.79	13.71±9.16	2.10±0.68	3.53±1.99	41.21±4.91	33.34±9.59	0.12±0.08	0.07±0.05	0.81±0.35	0.04±0.03
		3m	0.33±0.21	0.79±0.52	3.46±1.10	17.96±8.52	0.79±0.51	3.29±1.20	31.67±9.80	40.23±8.85	0.13±0.09	0.26±0.16	0.96±0.28	0.12±0.03
		6m	0.96±1.01	0.66±0.59	4.98±2.12	16.33±7.05	0.98±0.49	2.28±0.49	29.09±13.93	39.98±15.50	0.23±0.08	2.09±2.03	2.12±0.76	0.28±0.28
	Site 4	0m	0.08±0.11	1.66±0.44	2.08±0.84	10.09±5.23	2.87±1.14	2.58±1.81	43.44±11.39	36.02±5.58	0.14±0.07	0.23±0.28	0.74±0.22	0.05±0.02
		3m	1.66±1.38	0.99±0.59	2.76±0.66	13.33±4.17	1.55±1.06	2.16±2.07	41.85±15.76	34.28±9.60	0.16±0.05	0.22±0.23	0.92±0.29	0.12±0.06
		6m	0.70±1.35	0.28±0.23	3.60±1.24	10.05±4.09	1.51±0.80	1.91±1.47	39.18±17.94	40.97±11.92	0.11±0.07	0.15±0.27	1.34±0.27	0.18±0.14
Senecio cineraria	Site 1	0m	1.40±2.01	1.81±1.25	2.06±1.24	8.02±5.63	1.60±0.91	2.56±1.23	41.33±11.60	38.03±6.10	0.44±0.69	0.34±0.10	2.36±2.18	0.03±0.02
		3m	1.40±2.11	0.88±0.51	3.42±1.32	14.46±5.36	3.53±1.29	2.63±0.89	24.15±17.03	33.24±7.19	1.51±0.77	0.26±0.20	9.88±4.66	4.63±3.57
		6m	1.51±2.05	0.93±0.55	3.52±1.32	14.64±5.41	3.59±1.27	2.65±0.87	23.71±16.26	33.14±7.24	1.52±0.77	0.34±0.22	9.98±4.66	4.69±3.56
	Site 2	0m	2.63±0.96	2.55±0.53	3.66±2.57	15.15±8.14	2.15±0.84	3.88±1.69	30.95±7.90	34.85±8.74	0.60±0.33	0.26±0.06	3.27±1.59	0.05±0.04
		3m	0.35±0.49	0.98±0.78	7.10±2.78	27.92±12.22	0.36±0.54	0.77±0.98	29.54±10.91	23.55±3.89	1.03±0.47	0.22±0.09	8.03±4.56	0.13±0.10
		6m	0.11±0.24	0.65±0.38	10.56±1.03	42.66±4.78	0.02±0.02	0.44±0.41	11.02±2.63	16.28±2.16	1.71±0.68	0.33±0.27	16.15±8.64	0.06±0.08
	Site 3	0m	2.56±4.79	2.02±1.17	3.46±1.61	14.38±8.03	2.312.32±	3.33±2.73	36.61±8.39	30.368.14±	0.61±0.58	0.28±0.06	4.04±3.65	0.03±0.02
		3m	1.46±1.93	0.81±0.98	6.17±1.85	27.93±9.63	0.10±0.07	0.54±0.45	16.27±4.46	37.34±8.97	1.17±0.59	0.36±0.11	7.75±4.18	0.10±0.05
		6m	0.48±0.65	0.80±0.77	3.77±2.31	15.02±8.84	0.74±0.59	1.99±2.02	22.96±11.33	45.13±7.32	0.66±0.47	0.77±0.62	7.46±3.37	0.22±0.15
	Site 4	0m	2.86±2.62	4.65±4.52	2.40±1.25	8.26±4.50	2.07±0.90	4.74±2.36	38.24±10.27	31.44±4.53	0.42±0.29	0.82±0.55	3.84±3.37	0.25±0.18
		3m	4.16±3.29	2.81±1.23	1.23±0.48	4.07±1.91	2.40±1.30	6.05±1.16	44.45±2.49	32.68±4.76	0.31±0.11	0.22±0.15	1.45±0.65	0.14±0.08
		6m	0.09±0.21	1.50±0.85	5.20±1.17	20.34±4.16	3.362.27±	3.22±1.50	15.06±5.89	31.85±7.77	1.83±0.89	0.56±0.32	16.65±8.34	0.13±0.22

Values are mean (n = 5). Limits of detection (LD) Mn= 0.44; Fe= 5.41; Ni 2.60; Cu 0.47; Cr=1.83 Normalized wt %
